# Illuminating the future of DNA sequencing

**DOI:** 10.1186/gb4165

**Published:** 2014-02-25

**Authors:** Mick Watson

**Affiliations:** 1 Edinburgh Genomics, The Roslin Institute and Royal (Dick) School of Veterinary Studies, University of Edinburgh, Easter Bush, Midlothian EH25 9RG, UK

## The battle for humanity

Human (clinical) genome sequencing is the biggest potential market in DNA sequencing, and it is this market that all of the sequencing companies are striving to capture. In another article in this issue of *Genome Biology*, Neil Hall expresses some concern over the limitation of Illumina’s newly announced Hiseq X Ten platform to human (http://genomebiology.com/2014/15/2/104); indeed, at face value this does appear strange. The fact that Illumina have presented PhiX data from the X Ten confirms that there is no limitation inherent to the technology. The limitation is one of licensing. However, those involved in human genome sequencing will not be surprised by the move.

Human genome sequencing has been far cheaper than other genome sequencing for many years, and the X Ten is simply an extension of that pattern. The reason for this is Complete Genomics, a sequencing company whose technology is currently limited to human genomics. It’s cheaper to sequence a human genome with Complete Genomics than it is to sequence the same genome on your own Illumina instruments. Up until now, Illumina’s answer to this has been to sequence human genomes in house, matching Complete Genomics’ price. By my estimate, sequencing human genomes at Illumina’s in-house sequencing services is approximately 50% of the cost of sequencing the same genomes on your own Illumina instruments. Illumina are actually undercutting their own customers on human genome sequencing, and have been for years. I doubt this is a position they enjoy, but this is how they have chosen to compete with Complete Genomics.

So human genome sequencing has always been different, and the X Ten’s limitation is simply an extension of that.

## It’s just business

Imagine you have a business, making and selling shoes. You make all kinds of shoes: sports shoes, business shoes, ballet shoes, tap-dancing shoes, and you sell them all at £100 per pair. You dominate the market. Then a competitor comes to your attention - they also make shoes, but at present, they only make sports shoes. They sell their sports shoes at £50 per pair. What do you do? Your competitor only sells sports shoes - you have to compete, so reducing the cost of your sports shoes to £50 per pair is a no-brainer, but there is no competitor in the market for the other types of shoe. You can still sell those at £100 per pair. Would you also reduce the cost of all your other shoes? I don’t think you would; or if you did, then you wouldn’t be a very good businessman.

Illumina have a viable competitor in human genome sequencing, and so with the X Ten, they are competing with Complete Genomics for the human genome sequencing market. However, to expect them to reduce costs in other markets, and therefore reduce their profits, when they do not face any realistic competitor in those markets, is to expect Illumina to have poor business acumen. It is to expect Illumina to make a decision that would damage their business and reduce their profits. Illumina are a business, first and foremost. When working with Illumina I have always found them to be helpful and collaborative, but I am under no illusions: they act in this way because it benefits their business. As scientists, we cannot and should not expect companies to behave in any other way. They have an obligation to their shareholders to make a profit. None of us should be surprised by the X Ten’s human limitation. In fact, it is an entirely logical step.

For me, an interesting question is how the other markets react. For example, will those working in pig genomics (the pig genome is approximately the same size as the human genome) continue to pay $7,000 for a 30x genome when they know that their human counterparts can get the same for $1,000? Or will they say, ‘No, we will wait until the $1,000 pig genome is available’. If the other sequencing communities decide to hold onto their money, waiting for the X Ten license to be relaxed, then this will harm the current Hiseq 2500 market and may influence Illumina’s future strategy. Alternatively, should Complete Genomics become available to other species, this may also force Illumina’s hand.

## The Future

What has not been discussed up until now is the fact that Illumina have never released a system that is already at its peak performance; to put it simply, the Hiseq X will get better - run times will get faster, read lengths will get longer and read numbers will rise. The Hiseq X Ten can deliver the $1,000 genome *now*, but even cheaper human genomes are built in.

Of course, the Hiseq X license will relax at some point. At the moment, only human sequencing is available and only whole genome (using the TruSeq nano protocol). Eventually, however, the X system will be opened up - both to other types of sequencing (RNA-seq, exomes and so on) and to other species. Not only will we have the $1,000 genome, but the $100 exome, the $50 RNA-Seq. What Illumina will want to hear is that, as sequencing gets cheaper, researchers will either sequence deeper (more reads per sample) or broader (more samples at existing read depths). Users will eventually be able to purchase individual Hiseq X systems, rather than the current minimum buy of a cluster of ten, and the new machine is effectively the replacement for the Hiseq 2500. In 18 to 24 months, I anticipate Hiseq 2500 owners to trade them in for Xs - either that or find themselves undercut by other facilities.

It will be really interesting to see how Life Technologies responds, as manufacturers of the Ion Torrent sequencing platforms (the Personal Genome Machine and the Proton). Their key advantage is speed, with the Ion platforms carrying out the sequencing component in hours rather than days. However, the throughput and cost-per-base do not match current Illumina platforms, never mind the new ones. To remain a viable business, Life Technologies must respond. Both companies may also face competition from Roche, a global healthcare company who recently announced an intriguing agreement with Pacific Biosciences (http://investor.pacificbiosciences.com/releasedetail.cfm?ReleaseID=793199) to develop DNA sequencing products for clinical diagnostics. As a large multinational corporation, Roche have the financial clout to be a disruptive influence in the market. Having previously made moves to purchase Illumina, the renewed interest shown by Roche is an early signal of intent to move into human clinical sequencing.

## Competing interests

The author declares that he has no competing interests.

## Twitter

You can follow the author and discuss this column with him on Twitter at @BioMickWatson.

